# Impact of Sanitation on Rodent Pullulation and Plague Status in an Informal Settlement on the Outskirts of Mahajanga (Madagascar)

**DOI:** 10.3390/pathogens13110918

**Published:** 2024-10-22

**Authors:** Soanandrasana Rahelinirina, Zara Nomentsoa Razafiarimanga, Minoarisoa Rajerison, Medard Djedanem, Pascal Handschumacher, Ronan Jambou

**Affiliations:** 1 Plague Unit, Pasteur Institute of Madagascar, BP1274 Ambatofotsikely, Antananarivo 101, Madagascar; rahelinirina@pasteur.mg (S.R.); mino@pasteur.mg (M.R.); 2 Faculty of Sciences, University of Antananarivo, Antananarivo 101, Madagascar; razafiarimanga@gmail.com; 3 Centre D’étude et de Recherche Médicale et Sanitaire (EPICENTRE), Niamey BP 13330, Niger; medardndje@gmail.com; 4 SESSTIM, UMR 259 IRD, U1252 INSERM, Aix Marseille University, 13005 Marseille, France; p.handschumacher@unistra.fr; 5 Global Health Department, Pasteur Institute, 75015 Paris, France

**Keywords:** sanitation, rat, flea, plague, Mahajanga, Madagascar

## Abstract

Plague is a zoonotic disease caused by *Yersinia pestis*, and it is endemic in Madagascar. The plague cycle involves wild and commensal rodents and their fleas; humans are an accidental host. Madagascar is the country where plague burden is the highest. Plague re-emerged in Mahajanga, the western coast of Madagascar, in the 1990s and infected populations in the popular and insalubrious zones. Sanitation is considered a primary barrier to infection by excluding pathogens from the environment and reservoirs. Poor housing and hygiene and proximity to rodents and fleas in everyday life are major and unchanged risk factors of plague. The aim of this study was to measure the impact of sanitation on *Yersinia pestis* bacteria in human and small mammal reservoirs and flea vectors. This study was conducted on 282 households within 14 neighborhoods. Two sessions of sampling were conducted in 2013 and 2016. Small mammals were trapped inside and around houses using live traps. Fleas, blood and spleen were sampled to detect *Y. pestis* infection and antibodies and determine the level of plague circulation before and after the installation of sanitation in order to assess the impact of sanitation improvement on inhabitant health. Two major types of housing can be described, i.e., formal and informal (traditional), scattered in all the suburbs. Among the small mammals captured, 48.5% were *Suncus murinus*, and 70% of houses were infested. After sanitation, only 30% of houses remained infested, and most of them were located around the market. Fleas were mostly *Xenopsylla cheopis.* Before and after intervention, the overall prevalence of fleas was the same (index 4.5) across the 14 suburbs. However, the number of houses with fleas drastically decreased, and the flea index increased significantly in rodent-infested houses. Rodent abundance also decreased from 17.4% to 6.1% before and after intervention, respectively. A serology study highlights that plague is still circulating in Mahajanga, suggesting that small mammals maintain enzootic plague transmission in the city.

## 1. Introduction

Plague is a zoonotic bacterial disease associated with rodents and their fleas, caused by *Yersinia pestis*, a Gram-negative bacterium. Transmission of the disease is mainly caused by flea bites, but handling and eating an infected animal can cause human infection, as can aerosol transmission. The disease is often fatal in humans if left untreated. Plague continues to occur in several geographically distinct foci corresponding to a wide range of habitats in Asia, the Americas or Africa [1], both rural and urban [2,3]. Each plague focus has different characteristics, but they all share an epidemiological cycle involving mammals as reservoirs and fleas as vectors. Globally, the disease can affect over 200 species of mammals [4], and 60 species of fleas can transmit plague [5]. Wild animals appear to be involved in the epidemiology of most plague outbreaks and serve as important reservoirs for transmission of the pathogen to domestic animals and humans.

With 80% of the global human cases reported between 2013 and 2018 [1], Madagascar is the country most affected by plague in the world. As a disease of poverty, plague remains a major public health threat, in part because of its high epidemic potential and high lethality in some regions. It has also been observed that natural outbreaks tend to spread. In Madagascar, the incidence of human plague is highly seasonal, with the highest rate observed from September to April in the central highlands and from July to November in Mahajanga, the only coastal focus. The reservoirs of plague in Madagascar are *Rattus rattus*, *Rattus norvegicus* and *Suncus murinus* [6,7,8]. Two flea species have been identified as vectors, the oriental flea *Xenopsylla cheopis* and the endemic flea *Synopsyllus fonquerniei* [6].

On the northwest coast of Madagascar, the port of Mahajanga has been affected by the transmission of plague and cholera in recent decades. Plague reappeared in Mahajanga in 1991 after 60 years of silence [9,10] due to problems of hygiene, insecurity and long-term poverty [9,11]. The most recent plague outbreak occurred in 1999 in the city’s informal settlements, highlighting the geographical disparity between under-integrated and non-integrated neighborhoods. *Yersinia pestis* was isolated from humans, small mammals and fleas in Mahajanga during plague epidemics between 1991 and 1998. No human cases have been reported since 2000, but the bacterium is still circulating at low levels in small mammals.

Sanitation is considered a primary barrier to infection by eliminating reservoirs of the pathogen from the human environment. However, in the neighborhoods close to the Metzinger valley, access to sanitation is the main problem for most residents. These neighborhoods are characterized by insalubrity and an accumulation of waste. The insalubrity of the city has been highlighted during recent epidemics and has attracted the attention of many actors in the field of urban development and public health. In this type of tropical city, one of the reasons for the accumulation of waste is the low number of bins available in neighborhoods and the inequitable frequency of collection. Overloaded bins are used to dump rubbish, which is spread by wind and water and blocks drainage channels. In recent decades, as the population has grown, the city has moved closer to the Metzinger valley and beyond. Today, the flood-prone Metzinger valley separates the city from the urban plan. As a result, the outlying districts are characterized by a total lack of urban planning, narrow, irregular streets and a lack of sanitation infrastructure.

Due to the illegality of these settlements and the lack of a coherent urban strategy, the city of Mahajanga has not made the necessary investments to improve the sanitation of these neighborhoods. To address this situation, a major sanitation project supported by French regional cooperation has been implemented in the area since 2014. 

The ASSMA project (2012–2016) aimed to improve sanitation in the city of Mahajanga through both family-scale measures (installation of latrines with septic tanks) and collective measures (standpipes and collective latrines, particularly in the markets). Although the installation of family latrines, which required a modest initial financial investment on the part of the beneficiaries, got off to a difficult start (73 family latrines between 2013 and 2014), these initial achievements set an example for the rest of the inhabitants of the Metzinger valley, speeding up the development process from June 2015 onwards. By the end of the ASSMA program (2016), 378 family latrines had been built, benefiting 1134 families or 5670 people. The 23 public latrine blocks that have been built benefit almost 20,000 people in addition to the family facilities.

Household waste collection has also been improved, with pre-collection committees set up in neighborhoods and 14 additional metal skips with a storage capacity of 100 m^3^ having been brought into service. At the end of the chain, a treatment station with a capacity of 5 m^3^ of sludge/day was built to process the additional waste collected.

Finally, the Mahajanga urban community and the NGO «ENDA Océan Indien» mobilized the local population with 4114 people volunteering to clear more than 26 linear kilometers of wastewater drainage channels.

Through a series of interlocking measures addressing the various determinants of insalubrity in the city of Mahajanga at multiple levels, this program has been able to improve the living conditions of the population in the most disadvantaged spatial sub-space of this coastal city.

The evaluation of the impact of this project was planned to be carried out through a transversal clinical survey of the population, a serological survey for plague and leptospirosis and a survey of small mammal reservoirs and flea populations in houses. The structure of the households was also studied in order to define the main risk factors for rodent pullulation. Indeed, the presence of rubbish and the lack of collective and family hygiene in the area of the flooded Metzinger valley are favorable conditions for the pullulation of small mammals, known reservoirs of plague, leptospirosis and diarrhea [12]. Improving this health situation requires better knowledge of the pathogens present in the area and the risk factors that favor their spread. A cross-sectional study was carried out at the beginning and after two years of the project to assess the impact of the activities carried out.

## 2. Materials and Methods

### 2.1. Study Sites and Sanitation Program

**Figure 1:**
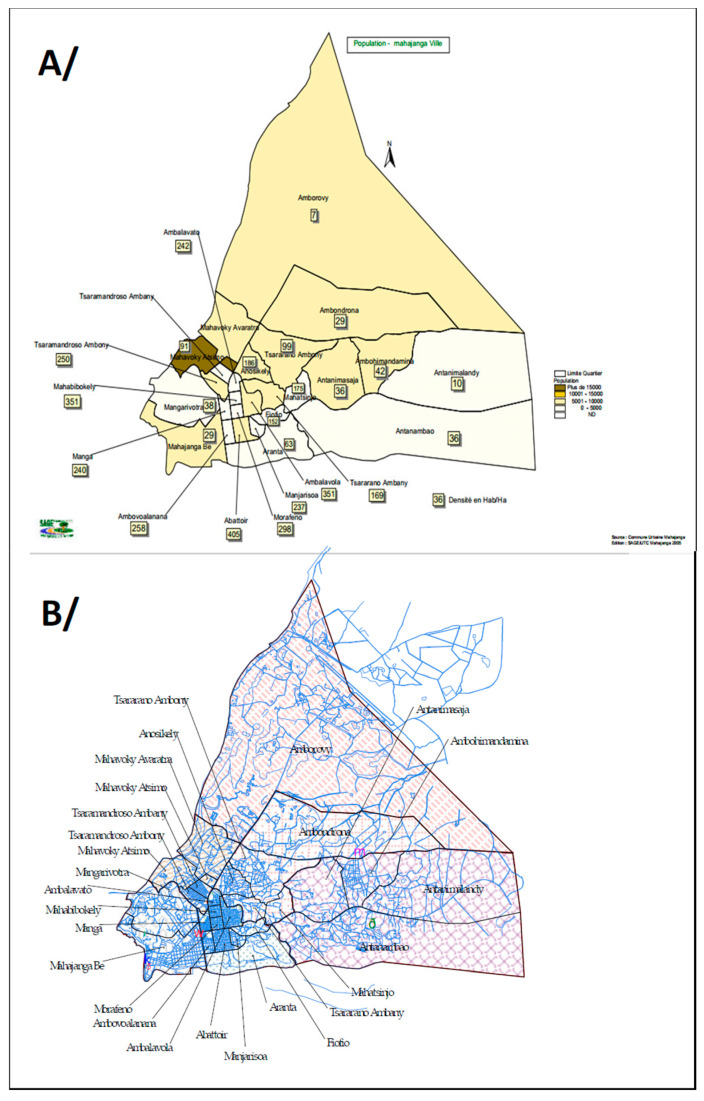
Map of Mahajanga city, showing the 14 suburbs investigated. (**A**): Administrative map of Mahajaunga, showing location of the 14 suburbs; (**B**): Map of the discharge culverts of the town showing very different densities in central and peripheral suburbs.

This study was conducted in 14 neighborhoods within the city of Mahajanga, the only coastal plague foci in northwestern Madagascar [12]. Five of the fourteen neighborhoods are located on either side of the Metzinger valley (a large canal built by Metzinger to collect surface water). Nine neighborhoods are located outside this valley (Figure 1).

Improperly disposed of waste provides harborage for rats. Improving sanitation is a crucial strategy for controlling rodents and can generate public health benefits by reducing the frequency and distribution of bacterial diseases and the population dynamics of rodents that are reservoirs for several zoonoses. In Mahajanga, the installation of latrines (to reduce dysentery disease), the cleaning of the canals and the organization of waste collection services (to reduce the availability of food and shelter for rodents) were carried out by the non-governmental organization Environment, Development and Action (NGO ENDA) between 2013 and 2017.

### 2.2. Household Description

Twenty houses were surveyed in each of the fourteen neighborhoods. Most of the houses are mainly made of metal sheets (roof and walls), and the floors are made of dirt cement. However, the structure of the floor, walls and roof was recorded for each house. The surroundings of each house were visually inspected by the team members to register the presence of garbage and surface water. The results of the qualitative inspection were scored from 0 (clean) to 3 (a lot of rubbish and garbage around the house).

### 2.3. Small Mammal and Flea Sampling

We collected the following plague risk indicators: rodents and fleas’ abundance and F1 antigen and IgG antibodies’ prevalence before and after sanitation. All animals were captured and handled in accordance with the guidelines of the American Society of Mammalogists [13] and European Parliament Directive 2010/63/EU (http://eur-lex.europa.eu/Lex-UriServ/LexUriServ.do?uri=OJ:L:2010:276:0033:0079:EN:PDF, accessed on 23 September 2024). The small mammals sampled were of introduced species (house mouse, black rat, brown rat and Asian house shrew), all of which are considered pests and therefore not protected in Madagascar. Each trapping campaign was validated by the national, regional and local health authorities.

Small mammals were trapped in the 14 suburbs (*fokontany* in Malagasy) during two sessions in August 2013 (pre-sanitation) and August 2016 (post-sanitation) using Besancon Technical Service (BTS) wire mesh live traps and Sherman box traps. During each trapping session, 20 houses were surveyed by *fokontany*. One BTS and one Sherman were placed inside each house. Each house was trapped for three consecutive nights during each trapping session (i.e., six traps per house). The traps were baited with onion and dried fish, set in the evening and checked each morning. The room in which the animals were caught was recorded.

Captured small mammals were euthanized by cervical dislocation and necropsied, i.e., weighed, measured and sexed. Species identification was based on morphological measurements. Fleas were removed from the small mammals by brushing the fur on the ventral and dorsal sides and preserved in 70% ethanol for identification using the taxonomic keys presented in Duchemin [14] to determine the flea index (average number of fleas per host). To describe plague exposure and infection in small mammals, blood samples were collected by cardiac punction, and serum was collected and frozen at +4 °C in the field and stored at −20 °C in the laboratory. They were used to assess IgG anti-F1 antibodies against *Y. pestis* by ELISA. Spleen samples were collected and preserved in Cary Blair transport medium (stored at ambient temperature) to test for the presence of *Y. pestis* F1 antigen using rapid diagnostic tests (RDTs) and to isolate *Y. pestis* strain by bacteriology.

Off-host fleas inside houses were trapped using light trap using candle placed in the middle of a plate containing soapy water, set in the middle of the room and lit by the homeowner when they turned off their own light at night. Fleas were attracted to the light, jumping into the soapy water. Fleas captured were collected using fine forceps and counted. All houses were examined for off-host fleas before sanitation (20 houses in each of the 14 neighborhoods), but after sanitation no free fleas were collected. Collection was conducted in the same house.

### 2.4. Laboratory Testing Biological Analysis

Spleen samples from small mammals were ground and tested for plague using F1 antigen lateral flow rapid test RDT and bacteriology culture. Sera samples collected from human and small mammals were tested to detect IgG antibodies against the capsule-like surface F1 antigen of *Yersinia pestis* using ELISA as previously described [15,16], with minor modification. Rapid Diagnostic Test used the same antigen. Spleen samples from rodents were ground and tested using the rapid diagnostic test (RDT) for F1 antigen detection based on lateral flow immunochromatography [15]. Samples that tested positive by RDT were analyzed through bacterial culturing for *Y. pestis* isolation using Cefsulodin–Irgasan–Novobiocin (CIN) agar media at 26–28 °C for 48 h [16].

The detection of anti-F1 IgG antibodies in rodents was performed by enzyme-linked immunosorbent assay (ELISA) as previously described (Dromigny et al., 1998), with minor modification. Detection was carried out on a plate coated with F1 antigen diluted in carbonate buffer, alongside a control plate coated with carbonate buffer alone to identify background readings. For the revelation step, an anti-rat IgG peroxidase conjugate (Sigma Aldrich, St Quentin Fallavier, France 1:15,000) and an anti-mouse IgG peroxidase conjugate (Sigma, 1:1500) were used for rodents and protein A peroxidase conjugate (Sigma, 1:5000) for shrews [17,18,19]. For ELISA, specificity and sensitivity were tested (99%, 94%) according Bezerra et al. [19].

### 2.5. Data Analysis

Samples were analyzed for each trapping site and for each trapping session to detect any differences in plague indicators. The proportion of fleas in the infested host was calculated. The flea index was calculated for each house and each trapping session as the total number of fleas divided by the total number of hosts. The abundance of each small mammal species at each site per trapping session was calculated by trapping success as the number of individuals trapped per trapping night. Host species richness and diversity were calculated for each site.

Correlation between household and rodent trapping was analyzed either by considering a house as positive if at least one mammal was trapped or by considering the number of rats/fleas trapped for positive houses only. Household typology was performed using multi-component analysis on Tanagra software (https://tanagra.software.informer.com/, accessed on 23 September 2024) with house descriptors as variables. Positive houses for rodents and fleas were added as additional variables. Identification of risk factor for rodent and flea infestation was performed using bivariate analysis on qualitative data and then by performing backward logistic regression with the presence of rodents in houses or of fleas (positive/negative) as variables to be explained.

## 3. Results

### 3.1. Structure of Houses

**Figure 2:**
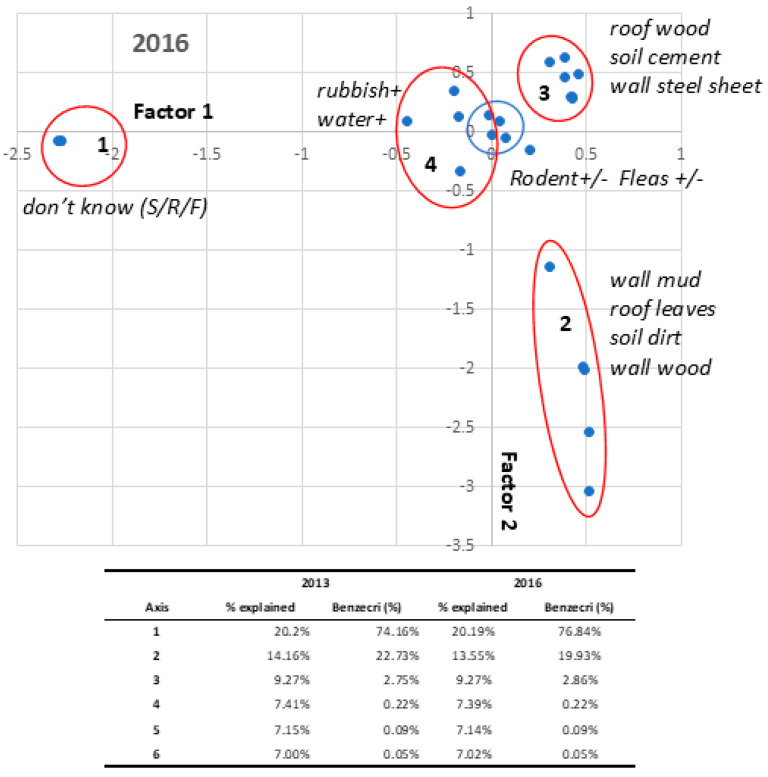
Multiple component analysis. Axes 1 and 2 are plotted. Four groups of houses can be identified: 1—houses with unknown status; 2—informal building; 3—houses with formal setting; 4—houses with rubbish and wastewater in the vicinity. Presence of rodent (yes/no) and or fleas (yes/no) is plotted on the same graph (blue circle). The table reports on the percent of variance supported by each axis (with or without Benzecri correction). The two first axes carry 100% of the information after correction.

Irrespective of the suburb, the structures of the houses were fairly homogeneous (Table 1). Most houses had a cement floor, walls of tin (or wood or brick) and a roof of tin or leaves. These are the typical building materials used in this region of Madagascar. However, for a better typology of housing, a multi-component analysis of the house descriptors was carried out in 2013 and 2016 (Supplementary Table S1, Table 2, Figure 2). No difference was found between the two years, and only the 2016 data are commented on. Five factors summarized almost all the information (Benzecri criteria: 100%). The first axis separates houses with known or unknown descriptors. The second axis separates two groups of houses, i.e., informal households with mud walls, earth floors, traditional roofs, etc., and the opposite, more formal habitat. The presence of rubbish or surface water around the house defined a third sub-group within the formal habitat. When the “neighborhood” is added as a supplementary variable, all the neighborhoods were plotted close to zero of the axes, suggesting that no difference can be seen in terms of habitat, all of them housing different types of households.

To assess the safety of the environment, rubbish and stagnant water around the houses were recorded during the trapping nights. Surprisingly, almost no improvement was observed for these descriptors from one session (2013) to another (2016, Table 1). This may be related to the process of self-enrolment of the residents in the project, but it may also be the perception of the environment around the house as part of the self or separate from it.

### 3.2. Small Mammal Density and Flea Infestation

A total of 394 small mammals representing three rodent species (*R. rattus*, *R. norvegicus*, *M. musculus*) and one shrew species (*Suncus murinus*) were captured during the two trapping seasons (Table 3). Shrews were the predominant small mammal with no difference between years (51.3% versus 49.6%), followed by *R. norvegicus*, which increased over the period (28.1% versus 38.4%). *R. rattus* was found at only five sites. The sites with the most shrews were those with less *R. norvegicus* (in 2013 Chi2 6.227, p 0.012).

The trapping success (over the whole houses) was 17.4% before sanitation (2013) and 6.1% after sanitation (2016, *p* < 0.05). In the same line, the percentage of positive houses decreases drastically from 78.7% to 30.6%. However, the density of mammals in positive houses did not decrease from one year to the next (1.3 versus 1.4). Interestingly, before sanitation, the number of positive houses and the number of rodents caught were quite homogeneous from one suburb to another, but after sanitation, most of the positive houses and rats were located mainly in two suburbs around the abattoir. 

During the two sessions, a total of 1792 fleas were collected from all mammals, and almost all were identified as belonging to *Xenopsylla cheopis*. The number of fleas collected from each host varied from 0.2 to 16.7. The global flea index was the same before and after sanitation (4.5 versus 4.6). However, this index varied significantly between suburbs, especially in 2016, with three suburbs averaging more than 10 fleas/rat (abattoir area). *R. norvegicus* was the most flea-infested species (one rat carrying 66 fleas). A total of 32 off-host fleas were collected before sanitation, belonging to two species: 27 *Ctenocephalides felis* (cat fleas) and 5 *X. cheopis* (rat fleas).

By ELISA, 3.8% of the 388 blood samples collected from mammals showed antibodies against *Yersinia pestis* (4.8% in 2013 and 1% in 2016) (Table 4). Using RDT, 2.8% of small mammals tested positive for plague in 2013. Seropositivity was higher in suburbs close to the Marolaka market, the historical focus of plague in Mahajanga. However, no difference in serology was found in suburbs according to their distance from the Metzinger valley.

### 3.3. Risk Factors for Rodents’ Infestation

#### 3.3.1. Bivariate Analysis

For this analysis, each category of roof, wall and floor was analyzed separately according to the presence of rodents or fleas in the house. In 2013, when a large proportion of houses were contaminated by rodents, no variable reached the significant threshold (chi-squared, *p* = 0.5) to explain this presence (Table 5). In 2016, a much lower number of houses were contaminated, and two parameters reached this threshold and were positively associated, i.e., palm-leaf roof (“satraka” traditional housing) for rodents and brick walls for fleas.

#### 3.3.2. Multivariate Analysis

The presence of rodents or fleas was first analyzed using multi-component typing of households (Figure 2). Presence/absence was introduced as a supplementary variable and visualized on the same graph (Supplementary Table S1, Table 2, Figure 2). Fleas and rodents were both grouped around zero for all axes in close relation to the presence of rubbish and surface water, suggesting a poor correlation with the household descriptors.

Between 2013 and 2016, no clear difference was observed in the typology of the houses nor in the cleanliness of the environment (waste and stagnant water). A logistic regression analysis was carried out using all the household descriptors and the presence of rodents or fleas as explanatory variables. They were included in a backward process, with an exclusion threshold of *p* > 0.2 (Table 6). Rodents were first considered as a whole, and then *R. norvegicus* and *S. murinus* were analyzed separately. Indeed, a clear dichotomy appeared between *Rattus* and *Suncus* in house colonization (in 2013 Chi2 6.227, p 0.012).

In 2013, due to the high contamination of living quarters by rodents, the logistic regression models were not significant with a poor classification rate (35 and 43% error for rodents+ and fleas+, respectively). However, traditional roofs (palm leaves) and cement floors increase the presence of rodents (not significant). Walls made of brick, steel or wood reduce the presence of fleas, while traditional roofs increase it (Table 6). In 2016, the number of houses with rodents (house+) or fleas (fleas+) decreased drastically. However, this decrease was not associated with a change in the structure of the houses or in the cleanliness of the environment. The logistic models became significant for the two variables to explain (*p* = 0.0009 and 0.023 for rodents+ and fleas+, respectively). The classification error rate improved to 30% for houses+ and 17% for fleas+. However, none of the descriptors seemed to be clearly related to the explanatory variables.

For *Rattus* in 2013, the presence of rubbish and soil in cement reduced the trapping of rodents in the house studied, possibly because they preferred to stay outside these houses. In 2016, this effect of the environment was no longer significant; soil in cement was still less favorable for rodents, but the quality of the walls was predominant. For the presence of *Suncus*, the soil in the floor and traditional roofs were the two main factors, although the presence of *Rattus* should be tested by itself (as competitors).

## 4. Discussion

Mahajanga is a fast-growing, medium-sized city on the west coast of Madagascar. This port has traditionally had close links with the Comoros archipelago and the island of Zanzibar. It is partly built on a flat area surrounded by hills. During the colonial period, a canal was built to collect waste and rainwater. However, the area along the canal is an area of rapidly growing informal settlements with very little urban renewal. Thirty years ago, a plague epidemic spread rapidly through the city from the Marolaka market and slaughterhouse. Cholera epidemics were also regularly recorded. In Madagascar, plague is usually transmitted in the highlands above 600 m, but in Mahajanga a new ecosystem [9,10,11] has been established. In order to improve sanitation in the suburbs of the city, a project supported by French regional cooperation was launched in 2012. The aim of the project was to improve access to latrines, rubbish bins and water supplies. The project was based on a new sanitation management system. Every household had to register for the project and contribute to the construction of latrines. The enrolment process was then quite slow, as the main objective was for residents to take ownership of the whole project for their suburbs. The evaluation of the impact of the projects was initially based on the economic sustainability of the system but also on health aspects. Malnutrition, intestinal parasites, leptospirosis and plague were the main concerns [12]. Household rodent infestation and the presence of fleas were also included as criteria for evaluating the effectiveness of the project for the whole city. No specific activities were carried out to control rodents and fleas in the home.

This study focuses on evaluating the impact of the project on rodent and flea infestation in households. Two rounds of surveys were conducted at the beginning of the project (2013) and after three years of activities (2016). In addition to describing the habitat of the area, three questions needed to be considered for the future of the project: (i) What are the factors associated with the presence of rodents and fleas in these neighborhoods? (ii) Is the plague still present and can it be reactivated? What has been the impact of the project on the overall health situation in the area? 

### 4.1. Organization of the Suburbs

Fourteen neighborhoods in the rapidly developing periphery of the city were surveyed. The administrative division is clear, and regular censuses are carried out. Habitat mapping shows two distinct types of construction: informal/traditional and urbanized. However, this dichotomy is not reflected in the geographical distribution, and both types exist in every neighborhood. The same goes for the presence of garbage or stagnant water. This justifies a comprehensive approach to all neighborhoods, and community assessment should be supported, with owners of formal habitats helping those with informal ones. Rehabilitation of some suburbs will not be the solution, as all are affected.

### 4.2. What Factor Are Related with the Persistence of Rodents?

In 2013, rats were trapped in almost 70% of houses, whereas three years later only a minority of houses still had rats. These houses were concentrated around the Marolaka market. Two rodent species share houses without mixing: *S murinus* and *R. norvegicus*. However, this reduction in the rodent population was not clearly linked to a change in the structure of the houses or to the cleanliness of the environment around the houses (water and waste). No specific control program was organized in this area either. The persistence of rodents in some houses could be supported by the local context. As in Madagascar, in rural Tanzania, altitude is associated with an increase in small mammals. However, more importantly, soil type and land-use patterns can regulate this abundance [20,21]. Phosphorus, slope aspect and altitude were significant predictors explaining the richness and abundance of small mammals [22]. These parameters are not related to the urban context, but as described by [23] “*poor disposal of animal and human food, irregular garbage collection, unauthorised garbage storage, lack of accessible dustbins, poor bulk waste management, ownership problems and structural deficiencies as major factors favouring rodent proliferation in the study areas*”. This means that information and self-registration by householders will be the main driver for improving suburban sanitation.

For fleas, the flea index did not vary between neighborhoods in 2013. It increased sharply in the neighborhoods that remained infested in 2016, reflecting a potential risk of resurgence of the plague. Two species were found separately: (i) *Ctenocephalides felis* from a large number of free fleas, and (ii) *X. cheopis*, known as a plague vector, was mainly found in rodents but five have been found free, which poses a major risk to humans. A difference in the timing of peak abundance between flea species has already been observed during and outside the plague season [24]. In the same line, Kessy et al. [25] observed two flea communities that varied between the dry, long rainy season and the short rainy season. Previous studies on fleas have shown the influence of abiotic factors on abundance, intensity and infestation rate [26]. It could therefore play an important role in a potential human plague epidemic. After the general improvement of the suburbs, special attention must be paid to the few houses that are still infested with rodents and fleas.

However, how can the increase in the flea index in 2016 be explained and controlled? In some countries, flea infestation was positively associated with the short rainy season and with rodent body weight. This effect of rodent body weight varies from country to country. In some places, the flea index increases in malnourished rats, while in others it increases in well-fed rats. Rat weight could therefore be a confounding variable with climate. Indeed, flea abundance was highest in dry years preceding wet years [25]. Increased rainfall and soil moisture in the previous year could create humid microclimates in rodent burrows, whereas low rainfall in the current year (in a rural setting) could predispose rodents to malnutrition and flea parasitism [25].

The persistence of fleas on the soil has also been described in other contexts, depending on local conditions. In rural areas, the presence of fleas was clearly influenced by land management [20], especially when the soil has a high pH [22]. In urban areas, a high *P. irritans* index was associated with traditional dirt floors covered with a plant fiber mat [27], but in Mahajanga, in more than 75% of houses, the soil was made with cement and *P. irritans* was not found. Overall, climate can drive plague epidemiology, as in British India [28]. However, special attention should be paid to the control of the oriental fleas *X. cheopis* in Mahajanga.

### 4.3. Is Plague Still Circulating?

In 2014, *Y. pestis* was isolated from rats in Mahajanga [20], with a higher seroprevalence in suburbs close to the Marolaka market, the historical focus of plague in Mahajanga. In this study, a higher prevalence of antibodies was found in Aranta, close to the Marolaka, where plague re-emerged in 1991. In humans (unpublished data), a lower seroprevalence was found in people under 10 years of age, who are not exposed to specific risks from handling dead rodents, compared with older people. These data suggest that *Y. pestis* is still circulating at low levels in rodents, even in the absence of human cases since 2000. The presence of free-living off-host fleas, particularly in 2016, poses a risk of human outbreaks in the future. Indeed, in Uganda, the *Xenopsylla* index increased prior to the onset of the annual plague, particularly in years with human cases [29]. Similarly, the concentration of rodents in a few places can increase the risk of an outbreak, as described by Carlson et al. [30] and by Kessy et al. [31], who reported an increase in domestic flea bites in rural areas during the dry season prior to plague transmission.

### 4.4. What Is the Impact of the Program?

The three-year project in Mahajanga appears to have resulted in a significant reduction in the number of rodents in many homes. The project did not specifically target rodents but waste management and sanitation. Education was also an important part of the activities. It is likely that this resulted in a better understanding by the population of the factors that encourage rodent infestation. However, rodents persisted in their historical foci, with a dramatic increase in the flea index, which may be associated with the spread of the disease from rodents to humans. The risk of an epidemic is therefore still present and may be increasing.

At the same time, no household descriptor was clearly found to be associated with the proliferation of rodents and fleas, compromising the strategy of habitat improvement. The abundance of fleas could be regulated more by climate than by sanitation. A specific program targeting rodents in highly contaminated houses could be implemented. Eisen RJ et al. in Uganda [32] described the efficacy of 6 days of intensive lethal trapping. However, rodent numbers returned to pre-treatment levels within eight weeks. Similarly, Rahelinirina et al. [33,34] found no evidence of an effect of the trapping strategy one month after the end of the program. A specific vector control program should be added in combination with sanitation. A community-based intervention could play an important role in reducing human–rodent–flea contact.

Overall, the type of household in Mahajanga was not a determinant of rodent and flea infestation. The sanitation program implemented in the area appears to have reduced the number of rodent- and flea-infested sites but has not eliminated the risk. In order to avoid the risk of an outbreak, a vector control program should then be added in a second phase of the project, targeting specifically contaminated houses and especially their surroundings to reduce the population of endemic fleas. This targeted strategy will be important to delay the emergence of insecticide resistance.

## Figures and Tables

**Table 1:** Typology of the houses investigated during the study in 2013 and 2016.

	2013																						2016																					
Suburbs	Soil				Walls							Roof					Waste Abondance **				Stagnant Water		soil				Walls							Roof					Waste Abondance **				Stagnant Water	
	NA *	Cement	Mud	Tiles	NA *	Tin Plates	Wood	Bricks	Cement	Cob	Tin + Wood	NA *	Tin Plates	Wood	Leaves	Palm	0	1	2	3	No	Yes	NA *	Cement	Mud	Tiles	NA	Tin Plates	Wood	Bricks	Cement	Cob	Tin + Wood	NA *	Tin Plates	Wood	Leaves	Palm	0	1	2	3	No	Yes
Abattoir	11	9			11	7		1	1			11	9					13	7		10	10	11	9			11	7		1	1			11	9					13	7		10	10
Ambalavola	4	15	1		4	14			3			4	16				13	5	2		13	7	8	13			8	10			3			9	12				14	5	2		14	7
Aranta	2	14	4		2	10	3	1	4			2	17			1		14	1	6	11	9	5	12	3		6	8	3	1	2			5	14			1		14	1	5	11	9
Fiofio	4	12	4		4	4	5		8			4	11			5	19		1		10	10	11	7	2		11	1	2		6			11	7			2	16	2	1	1	10	10
Mahatsinjo		11	8	1		14	3		1		2		17			3	10	6	2	2	19	1		11	8	1		14	3		1		2		17			3	10	6	2	2	19	1
Mahavoky Atsimo		19	1			11	3	6					17		3		17	1	2		15	5		19	1			11	3	6					17		3		17	1	2		14	6
Mahavoky Nord	6	14			6	7	1	6				6	13		1		2	18			19	1	6	14			6	7	1	6				6	13		1		2	18			19	1
Manga	1	17	2		2	13	1	3	1			2	17		1		19		1		17	3	1	17	2		2	13	1	3	1			2	17		1		19		1		17	3
Manjarisoa		18	2			13	5	2					18		2		17	2	1		18	2		18	2			13	5	2					18		2		17	2	1		18	2
Tsaramandroso Ambany		17	3			17	2		1	1			18		1	2	14	7	3	2	18	3		17	3	1		17	2		1	1			18		1	2	13	6	1	1	18	3
Tsaramandroso Ambony	2	17	1		2	17	1					2	18				17	2	1		17	3	2	17	1		2	17	1					2	18				17	2	1		17	3
Tsararano ambany	2	17	2		2	13	2		3	1		2	15	1		2	2	11	5	2	16	4	4	14	2		4	11	2		2	1		4	13	1		2	2	11	5	2	16	4
Tsararano Ambony	2	18			2	11	1	5	1			2	17		1		17		2	1	18	2	5	15			5	8	1	5	1			5	14		1		17		2	1	18	2
Tsararano Anosikely	1	17	2		1	14		2	3			1	19				1	17	3		20		1	17	2		1	14		2	3			1	19					17	3		20	
Total	**35**	**215**	**30**	**1**	**36**	**165**	**27**	**26**	**26**	**2**	**2**	**36**	**222**	**1**	**9**	**13**	148	96	31	13	221	60	**54**	**200**	**26**	**2**	**56**	**151**	**24**	**26**	**21**	**2**	**2**	**56**	**206**	**1**	**9**	**10**	144	97	29	12	221	61

* Don't know. ** 0 = no waste; 1 = few +/−; 2 = present ++; 3 = very abondant +++.

**Table 2:** Multi-component analysis of the household descriptors (factors 1 and 2).

	**2013**				**2016**			
**Descriptors**	**Factor_1**	**Factord_2**	ctr_1 ** %	ctr_2 ** %	**Factor_1**	**Factord_2**	ctr_1 ** %	ctr_2 ** %
**wall = steel**	0.42255	−0.3164	3.316	2.653	0.42841	0.28304	3.454	2.246
**wall = wood**	0.55199	2.46639	0.937	26.693	0.51047	−2.54128	0.74	27.33
wall = DN *	−2.26564	0.11636	27.325	0.103	−2.26737	−0.0736	27.385	0.043
**wall = cement**	0.37413	−0.49398	0.397	0.988	0.38948	0.45658	0.431	0.882
**wall = brick**	0.45134	−0.46093	0.602	0.896	0.45426	0.49255	0.611	1.069
**wall = mud**	0.49906	1.84521	0.059	1.149	0.47993	−1.98597	0.055	1.391
**roof = steel**	0.42525	−0.3032	4.535	3.289	0.42975	0.29723	4.635	3.302
**roof = leaves**	0.56121	3.02294	0.745	30.845	0.5152	−3.04346	0.628	32.666
roof = DN *	−2.26564	0.11636	27.325	0.103	−2.26737	−0.0736	27.385	0.043
**roof = wood**	0.28839	−0.70802	0.02	0.169	0.30987	0.58443	0.023	0.12
**Soil = cement**	0.41434	−0.32345	4.123	3.584	0.42037	0.29728	4.267	3.179
**Soil = dirst**	0.52699	2.05495	0.986	21.381	0.49074	−2.01847	0.827	20.834
Soil = DN *	−2.28811	0.11887	27.25	0.105	−2.28987	−0.07625	27.31	0.045
**Soil = tile**	0.3778	−0.60897	0.034	0.125	0.38362	0.62227	0.035	0.137
**rubish = +++**	0.32718	1.37644	0.152	3.837	0.30958	−1.14019	0.136	2.751
**rubish = ++**	−0.48791	−0.33715	0.817	0.556	−0.4422	0.08887	0.694	0.042
**rubish +/−**	−0.19516	−0.33262	0.433	1.793	−0.20077	0.34068	0.453	1.944
**rubish = 0**	0.20393	0.17741	0.699	0.754	0.20158	−0.15034	0.683	0.566
**water = yes**	−0.16692	0.27791	0.194	0.769	−0.16712	−0.32685	0.195	1.111
**water = no**	0.04476	−0.07453	0.052	0.206	0.04482	0.08766	0.052	0.298
**Supplementary variables**								
**house+ = neg**	−0.03894	−0.00391			0.07532	−0.0565		
**house+ = pos**	0.02244	0.00225			−0.17485	0.13116		
**flea+ = neg**	0.04681	−0.03048			0.00261	−0.02892		
**flea+ = pos**	−0.05065	0.03298			−0.01289	0.14273		

* don’t know/** contribution of the descriptor on the factor.

**Table 3:** Collection of rodents and fleas in the houses during this study in 2013 and 2016.

		2013										2016									
Fokontani	Nbr Houses	Nbr Houses+	Nbr Rats	Trap Success * (%)	Density Rats/House+ **	Nbr Fleas	Nrb Flea/Rat+	Sun. Mur.	Rat.Norv.	Rat.Rat.	Mus.Mus.	Nbr Houses+	Nbr Rats	Trap Succes * (%)	Density Rats/House+ **	Nbr Fleas	Nrb Fleas/Rat+	Sun. Mur.	Rat.Norv.	Rat.Rat.	Mus.Mus.
Abattoir	20	17	16	13.3	0.9	54	3.4	12	4	0	0	6	6	2.5	1.0	50	**16.7**	3	3	0	0
Ambalavola	21	19	15	11.7	0.8	107	7.6	1	**11**	0	3	4	4	2.5	1.0	37	**12.3**	2	2	0	0
Aranta	20	16	15	13.3	0.9	56	3.5	3	8	0	4	3	3	2.5	1.0	39	**13.0**	0	3	0	0
Fiofio	20	16	27	21.7	1.7	247	**9.5**	**12**	**11**	0	4	6	7	4.2	1.2	38	7.6	4	2	1	0
Mahatsinjo	20	18	**34**	**27.5**	1.9	159	4.8	**17**	5	4	8	**8**	**14**	**11.7**	1.8	32	2.3	7	1	6	0
Mahavoky Atsimo	20	15	20	7.5	1.3	79	4.2	**12**	4	0	4	7	9	7.5	1.3	26	2.9	4	5	0	0
Mahavoky Nord	20	13	26	15.8	2.0	84	3.2	**17**	5	1	3	3	4	3.3	1.3	32	8.0	3	0	0	1
Manga	20	16	16	13.3	1.0	66	4.1	3	**12**	0	1	**8**	**9**	5.8	1.1	39	5.6	4	3	0	0
Manjarisoa	20	18	22	18.3	1.2	68	3.1	**14**	4	1	3	2	2	0	1.0	0	0.0	2	0	0	0
Tsaramandroso Ambany	21	15	18	15	1.2	160	**8.9**	6	7	0	5	**7**	**14**	10.8	2.0	99	7.6	9	5	0	0
Tsaramandroso Ambony	20	15	21	16.7	1.4	45	2.3	**17**	2	0	2	5	7	5.8	1.4	47	6.	2	5	0	0
Tsararano ambany	20	17	25	20.8	1.5	68	2.7	**17**	3	2	3	4	8	5	2.0	1	0.2	5	1	0	2
Tsararano Ambony	20	13	22	17.5	1.7	71	3.4	7	7	2	6	**12**	**14**	11.7	1.2	42	3.0	6	7	1	0
Tsararano Anosikely	20	14	21	16.7	1.5	50	2.5	**15**	1	0	5	**11**	**16**	11.7	1.5	23	1.6	13	8	8	0
Total général	**282**	**222**	**298**	**17.4**	**1.3**	**1314**	**4.5**	**153**	**84**	**10**	**51**	**86**	**117**	**6.1**	**1.4**	**505**	**4.7**	**64**	**45**	**16**	**3**

* number of positive traps over the whole number of traps used; ** for positive houses only.

**Table 4:** Serology and antigen detection of *Y. pestis* in rats.

Suburbs	2013							2016			
	ELISA			RDT *		Rodents	Trapping Nights	ELISA		Rodents	Trapping Nights
	NA	Neg	Pos	Neg	Pos			Neg	Pos		
Abattoir	1	15		16		16	120	2		2	120
Ambalavola		14		14		14	120	3		3	120
Aranta		13	2	14	1	15	120	3		3	120
Fiofio		24	2	26		26	120	4		4	120
Mahatsinjo		30	3	32	1	33	120	13	1	14	120
Mahavoky Atsimo		16	3	19		19	120	7		7	120
Mahavoky Nord		26		26		26	120	4		4	120
Manga	1	15		16		16	120	7		7	120
Manjarisoa		22		20	2	22	120			0	120
Tsaramandroso Ambany	1	16	1	18		18	120	11		11	120
Tsaramandroso Ambony		20		20		20	120	7		7	120
Tsararano ambany	1	24		25		25	126	6		6	126
Tsararano Ambony	1	19	2	22		22	120	14		14	120
Tsararano Anosikely		19	1	20		20	120	14		14	120
Total	5	273	14	288	4	292	1686	95	1	96	1686

* RDT rapide diagnostic test.

**Table 5:** Bivariate analysis between presence of rodents and fleas and house descriptors.

		Houses with Rodents				Houses with Fleas				Total Houses
Types		NO (%)	Yes (%)	Khi^2^	*p*	NO (%)	Yes (%)	Khi^2^	*p*	(% *)
2013										
Soil	unknwon	14 (4.96)	22 (7.8)	0.167	0.683	17 (6.02)	19 (6.73)	0.203	0.653	36 (12.76)
	tiles	1 (0.35)	0 (0)	1.718	0.19	1 (0.35)	0 (0)	0.922	0.337	1 (0.35)
	cement	74 (26.24)	141 (50)	2.354	0.125	110 (39)	105 (37.23)	0.338	0.561	215 (76.24)
	mud	14 (4.96)	16 (5.67)	1.381	0.24	18 (6.38)	12 (4.25)	0.834	0.361	30 (10.63)
walls	unknown	23 (8)	33 (12)	0.52	0.46	26 (9)	30 (11)	0.90	0.34	56 (20)
	steel plate	56 (20)	95 (34)	0.006	0.93	83 (29)	68 (24)	1.05	0.30	151 (54)
	wood	8 (3)	16 (6)	0.14	0.70	13 (5)	11 (4)	0.043	0.83	24 (9)
	brick	8 (3)	18 (6)	0.45	0.49	14 (5)	12 (4)	0.033	0.85	26 (9)
	cement	9 (3)	12 (4)	0.34	0.55	11 (4)	10 (4)	0.0006	0.98	21 (7)
	mud	0 (0)	2 (1)	1.17	0.27	0 (0)	2 (1)	2.19	0.138	2 (1)
	steel and wood	0 (0)	2 (1)	1.17	0.27	0 (0)	2 (1)	2.19	0.138	2 (1)
roof	unknown	15 (5.31)	21 (7.44)	0.406	0.524	18 (6.38)	18 (6.38)	0.075	0.784	36 (12.76)
	steel plate	81 (28.72)	141 (50)	0.069	0.792	118 (41.84)	104 (36.87)	0.44	0.507	222 (78.72)
	wood	1 (0.35)	0 (0)	1.718	0.19	1 (0.35)	0 (0)	0.922	0.337	1 (0.35)
	plastic	2 (0.7)	7 (2.48)	0.858	0.354	3 (1.06)	6 (2.12)	1.316	0.25	9 (3.19)
	leaves	4 (1.41)	9 (3.19)	0.21	0.64	6 (2.12)	7 (2.48)	0.195	0.659	13 (4.6)
Total (2013)		103 (36.52)	179 (63.47)			146 (51.77)	136 (48.22)			282 (100)
2016										
Soil	unknwon	34 (12.05)	20 (7.09)	1.508	0.219	46 (16.31)	8 (2.83)	0.165	0.685	54 (19.14)
	tiles	140 (49.64)	60 (21.27)	0.007	0.935	165 (58.51)	35 (12.41)	0.344	0.558	200 (70.92)
	cement	21 (7.44)	5 (1.77)	1.619	0.203	22 (7.8)	4 (1.41)	0.034	0.854	26 (9.21)
	mud	2 (0.7)	0 (0)	0.869	0.351	2 (0.7)	0 (0)	0.403	0.526	2 (0.7)
Walls	unknown	36 (12.76)	20 (7.09)	1.03	0.31	47 (16.66)	9 (3.19)	0.018	0.894	56 (19.85)
	steel plate	106 (37.58)	45 (15.95)	0.018	0.894	126 (44.68)	25 (8.86)	0.003	0.957	151 (53.54)
	wood	19 (6.73)	5 (1.77)	1.079	0.299	22 (7.8)	2 (0.7)	1.312	0.252	24 (8.51)
	brick	16 (5.67)	10 (3.54)	0.941	0.332	18 (6.38)	8 (2.83)	4.101	0.043	26 (9.21)
	cement	18 (6.38)	3 (1.06)	2.709	0.1	19 (6.73)	2 (0.7)	0.833	0.36	21 (7.44)
	mud	1 (0.35)	1 (0.35)	0.377	0.539	2 (0.7)	0 (0)	0.403	0.526	2 (0.7)
	steel and wood	1 (0.35)	1 (0.35)	0.377	0.539	1 (0.35)	1 (0.35)	1.611	0.204	2 (0.7)
Roof	unknown	35 (12.41)	21 (7.44)	1.797	0.18	47 (16.66)	9 (3.19)	0.018	0.894	56 (19.85)
	steel plate	145 (51.41)	61 (21.63)	0.102	0.749	171 (60.63)	35 (12.41)	0.058	0.81	206 (73.04)
	wood	1 (0.35)	0 (0)	0.433	0.511	1 (0.35)	0 (0)	0.201	0.654	1 (0.35)
	plastic	6 (2.12)	3 (1.06)	0.045	0.832	6 (2.12)	3 (1.06)	1.859	0.173	9 (3.19)
	leaves	10 (3.54)	0 (0)	4.473	0.034	10 (3.54)	0 (0)	2.074	0.150	10 (3.54)
Total (2016)		197 (69.85)	85 (30.14)			235 (83.33)	47 (16.66)			282 (100)

* percent for 282 houses.

**Table 6:** Backward logistic regression between presence of rodents or fleas and house descriptors.

	**2013**				**2016**					**2013**			**2016**	
	**Rodents**		***R. norvegicus***	***S. murinus***	**Rodents**		***R. norvegicus***	***S. murinus***		**Fleas**			**Fleas**	
descriptors selected (*p* < 0.2)	5		3	3	5		4	1		9			3	
Positive value	presence		presence	presence	presence		presence	presence		presence flea			presence flea	
nbr houses included	282		280	280	282		280	280		282			282	
classification Error rate	35%		24%	36%	30%		13%	16%		43%			17%	
Chi−2 (d.f.)/*p* value	8.1884/0.14			17.428/0.0006	20.71/0.0009		9.26/0.054	2.76/0.09		11.3/0.253			9.15/0.027	
AIC Intercept/Model	373.28/375.1			372.47/361.04	347.20/336.49		224.96/223.69	252.87/252.1		392.42/399.08			256.11/252.9	
**Attribute**	**Coef.**	**Std-dev**	**Wald**	**Signif**	**Attribute**	**Coef.**	**Std-Dev**	**Wald**	**Signif**	**Attribute**	**Coef.**	**Std-Dev**	**Wald**	**Signif**
**Rodents**					**Rodents**					**2013**				
constant	−1.836	1.198	2.350	0.1253	constant	0.749	0.145	26.761	**0**	constant	−1.579	1.193	1.751	0.186
soil unknown	2.241	1.246	3.234	0.0721	lot of rubish	−21.055	8825.960	0	0.998	soil unknown	1.662	1.240	1.797	0.18
soil cement	0.844	0.429	3.878	0.0489	roof traditional	−33.341	6469.006	0	0.996	soil cement	0.708	0.449	2.488	0.115
roof steel plate	1.621	1.157	1.963	0.1611	wall cement	−1.014	0.646	2.461	0.117	wall steel plate	−0.950	0.472	4.054	0.044
roof leaves	2.570	1.411	3.315	0.0686	wall mud	20.051	6452.521	0	0.998	wall wood	−1.311	0.696	3.554	0.059
roof traditional	2.296	1.305	3.097	0.0784	some rubish	0.493	0.407	1.467	0.226	wall brick	−1.016	0.600	2.871	0.09
***R. norvegicus***					***R. norvegicus***					wall cement	−0.927	0.631	2.159	0.142
constant	0.174	0.675	0.066		constant	−1.935	0.381	25.805	0	roof steel plate	1.684	1.196	1.984	0.159
soil cement	−0.932	0.434	4.619	0.0316	soil cement	−1.025	0.480	4.568	0.033	roof leaves	3.055	1.442	4.486	0.034
roof steel plate	0.954	0.488	3.816	0.0508	wall steel plates	1.107	0.491	5.078	0.024	roof traditional	2.346	1.333	3.096	0.079
no rubish	−1.375	0.640	4.614	0.0317	wall bricks	1.525	0.696	4.806	0.028	**2016**				
some rubish	−1.457	0.657	4.923	0.0265	wall steel + wood	2.960	1.495	3.918	0.048	constant	−1.678	0.180	86.685	0
lot rubishes	−1.808	0.781	5.364	0.0206						wall wood	−1.906	1.175	2.632	0.105
***S. murinus***					***S. murinus***					wall brick	0.772	0.472	2.681	0.102
constant	−0.483	0.132	13.366	0.0003	constant	−1.553	0.166	87.630	0	roof leaves	2.319	1.193	3.780	0.052
soil dirt	−2.287	0.797	8.241	0.0041	soil dirt	−1.086	0.751	2.093	0.148					
roof traditional	2.681	0.949	7.976	0.0047										

## Data Availability

Data can be obtained from the first author, after reasonable demand.

## Supplementary Materials

The following supporting information can be downloaded at: https://www.mdpi.com/article/10.3390/pathogens13110918/s1, Table S1: Data related to axis of the multicomponent factorial analysis.
