# Supplementary material for: Impact of Sanitation on Rodent Pullulation and Plague Status in an Informal Settlement on the Outskirts of Mahajanga (Madagascar)
Source: Pathogens. 2024 Oct 22;13(11):918. doi: 10.3390/pathogens13110918 (PMC11597497; doi:10.3390/pathogens13110918)
Supplement: Supplementary file 1 [file pathogens-13-00918-s001.zip › pathogens-3192282-supplementary.pdf]

Axis	2013 % explained	2013 Benzecri (%)	2016 % explained	2016 Benzecri (%)
1	20,20%	74,16%	20,19%	76,84%
2	14,16%	22,73%	13,55%	19,93%
3	9,27%	2,75%	9,27%	2,86%
4	7,41%	0,22%	7,39%	0,22%
5	7,15%	0,09%	7,14%	0,09%
6	7,00%	0,05%	7,02%	0,05%

Descriptors	Mass	Sq.Dist	Inertia	coord_1	coord_2	coord_3	coord_4	coord_5	ctr_1 %	ctr_2 %	ctr_3 %	ctr_4 %	ctr_5 %
**2013**													
3_wall = steel	0,1125	0,7771	0,0875	0,42255	-0,3164	-0,21891	**-0,4767**	0,21458	3,316	2,653	1,939	11,503	2,417
3_wall = wood	0,0186	9,7308	0,1814	0,55199	**2,46639**	0,08586	0,0112	-0,27735	0,937	26,693	0,049	0,001	0,669
3_wall = NSP	0,0323	5,2	0,1677	**-2,26564**	0,11636	-0,07827	0,03883	-0,0583	27,325	0,103	0,071	0,022	0,051
3_wall = cement	0,0172	10,625	0,1828	0,37413	-0,49398	**2,26852**	**1,0285**	0,40959	0,397	0,988	31,832	8,186	1,346
3_wall = brick	0,0179	10,16	0,1821	0,45134	-0,46093	-0,66585	**1,5213**	**-1,66896**	0,602	0,896	2,857	18,655	23,279
3_wall = mud	0,0014	138,5	0,1986	0,49906	1,84521	-1,06982	**5,04356**	**4,01984**	0,059	1,149	0,59	16,404	10,804
3_roof = steel	0,152	0,316	0,048	0,42525	-0,3032	-0,07722	-0,07603	0,03804	4,535	3,289	0,326	0,395	0,103
3_roof = leaves	0,0143	12,95	0,1857	0,56121	**3,02294**	0,19488	0,4497	-0,06045	0,745	30,845	0,196	1,304	0,024
3_roof = NSP	0,0323	5,2	0,1677	**-2,26564**	0,11636	-0,07827	0,03883	-0,0583	27,325	0,103	0,071	0,022	0,051
3_roof = wood	0,0014	138,5	0,1986	0,28839	-0,70802	**7,99727**	2,68863	-2,11544	0,02	0,169	32,967	4,661	2,992
3_Soil = cement	0,1455	0,3744	0,0545	0,41434	-0,32345	0,01668	-0,02261	-0,10874	4,123	3,584	0,015	0,033	0,802
3_Soil = dirst	0,0215	8,3	0,1785	0,52699	**2,05495**	-0,1833	-0,03234	0,41045	0,986	21,381	0,26	0,01	1,69
3_Soil = NSP	0,0315	5,3409	0,1685	**-2,28811**	0,11887	-0,07295	0,033	-0,04504	27,25	0,105	0,06	0,015	0,03
3_Soil = tile	0,0014	138,5	0,1986	0,3778	-0,60897	2,66099	2,05444	**5,87139**	0,034	0,125	3,65	2,722	23,048
3_rubish = c	0,0086	22,25	0,1914	0,32718	**1,37644**	0,43502	**-1,72266**	0,79165	0,152	3,837	0,585	11,482	2,514
3_rubish = b	0,0208	8,6207	0,1792	-0,48791	-0,33715	**1,20753**	**-1,09464**	-0,32314	0,817	0,556	10,898	11,204	1,012
3_rubish = a	0,0688	1,9063	0,1312	-0,19516	-0,33262	-0,28685	0,3758	**0,7312**	0,433	1,793	2,036	4,371	17,158
3_rubish = n	0,1018	0,9648	0,0982	0,20393	0,17741	-0,08945	0,11507	**-0,49524**	0,699	0,754	0,293	0,606	11,643
3_water = p	0,0423	3,7288	0,1577	-0,16692	0,27791	**0,76567**	**-0,59016**	-0,12118	0,194	0,769	8,915	6,626	0,29
3_water = n	0,1577	0,2682	0,0423	0,04476	-0,07453	-0,20534	0,15827	0,0325	0,052	0,206	2,391	1,777	0,078
**2016**													
6_wall = steel	0,114	0,7547	0,086	0,42841	0,28304	0,22261	**0,48429**	-0,18442	3,454	2,246	2,03	12,05	1,811
6_wall = wood	0,0172	10,625	0,1828	0,51047	**-2,54128**	-0,05558	-0,07841	0,3902	0,74	27,33	0,019	0,048	1,224
6_wall = NSP	0,0323	5,2	0,1677	**-2,26737**	-0,0736	0,0693	-0,04239	0,0573	27,385	0,043	0,056	0,026	0,049
6_wall = cement	0,0172	10,625	0,1828	0,38948	0,45658	**-2,29544**	**-0,88607**	-0,44548	0,431	0,882	32,58	6,089	1,595
6_wall = brick	0,0179	10,16	0,1821	0,45426	0,49255	0,61709	**-1,69261**	**1,53311**	0,611	1,069	2,453	23,143	19,677
6_wall = mud	0,0014	138,5	0,1986	0,47993	-1,98597	1,24233	**-4,81579**	**-5,12864**	0,055	1,391	0,795	14,988	17,616
6_roof = steel	0,152	0,316	0,048	0,42975	0,29723	0,07278	0,07276	-0,02747	4,635	3,302	0,289	0,363	0,054
6_roof = leaves	0,0143	12,95	0,1857	0,5152	**-3,04346**	-0,12325	-0,40942	0,01555	0,628	32,666	0,078	1,083	0,002
6_roof = NSP	0,0323	5,2	0,1677	**-2,26737**	-0,0736	0,0693	-0,04239	0,0573	27,385	0,043	0,056	0,026	0,049
6_roof = wood	0,0014	138,5	0,1986	0,30987	0,58443	**-8,04117**	-2,66414	1,46704	0,023	0,12	33,318	4,587	1,441
6_Soil = cement	0,1462	0,3676	0,0538	0,42037	0,29728	-0,02687	0,0222	0,12236	4,267	3,179	0,038	0,032	1,023
6_Soil = dirst	0,0208	8,6207	0,1792	0,49074	**-2,01847**	0,27473	0,00211	-0,49412	0,827	20,834	0,564	0	2,371
6_Soil = NSP	0,0315	5,3409	0,1685	**-2,28987**	-0,07625	0,06473	-0,03474	0,04514	27,31	0,045	0,048	0,017	0,03
6_Soil = tile	0,0014	138,5	0,1986	0,38362	0,62227	-2,66731	-1,53068	**-6,30889**	0,035	0,137	3,666	1,514	26,657
6_rubish = c	0,0086	22,25	0,1914	0,30958	-1,14019	-0,32946	**2,04025**	-0,3182	0,136	2,751	0,336	16,141	0,407
6_rubish = b	0,0215	8,3	0,1785	-0,4422	0,08887	**-1,17419**	**0,98141**	0,34894	0,694	0,042	10,656	9,337	1,223
6_rubish = a	0,0681	1,9368	0,1319	-0,20077	0,34068	0,31293	-0,24241	**-0,70629**	0,453	1,944	2,397	1,804	15,87
6_rubish = n	0,1018	0,9648	0,0982	0,20158	-0,15034	0,06655	-0,21758	0,42569	0,683	0,566	0,162	2,172	8,617
6_water = p	0,0423	3,7288	0,1577	-0,16712	-0,32685	**-0,73663**	0,52175	0,10655	0,195	1,111	8,248	5,19	0,224
6_water = n	0,1577	0,2682	0,0423	0,04482	0,08766	0,19755	-0,13992	-0,02858	0,052	0,298	2,212	1,392	0,06

Supplementary variables	coord_1	coord_2	coord_3	coord_4	coord_5	F1 v.test	F2 v.test	F3 v.test	F4 v.test	F5 v.test
**2013**										
3_house+ = n	-0,03894	-0,00391	0,06653	-0,05253	0,0136	-0,493	-0,049	0,842	-0,665	0,172
3_house+ = p	0,02244	0,00225	-0,03834	0,03027	-0,00784	0,493	0,049	-0,842	0,665	-0,172
3_flea+ = n	0,04681	-0,03048	-0,02203	0,00081	-0,00643	0,812	-0,529	-0,382	0,014	-0,112
3_flea+ = p	-0,05065	0,03298	0,02383	-0,00088	0,00696	-0,812	0,529	0,382	-0,014	0,112
**2016**										
6_house+ = n	0,07532	-0,0565	-0,05634	0,01607	-0,03629	1,913	-1,435	-1,431	0,408	-0,922
6_house+ = p	-0,17485	0,13116	0,13079	-0,03731	0,08424	-1,913	1,435	1,431	-0,408	0,922
6_flea+ = n	0,00261	-0,02892	-0,03237	0,02437	-0,02779	0,097	-1,071	-1,199	0,903	-1,03
6_flea+ = p	-0,01289	0,14273	0,15977	-0,1203	0,13719	-0,097	1,071	1,199	-0,903	1,03
